# Editorial: Metabolic Disturbances in Mental Illness: Neuropathogenetic Mechanisms and Therapeutic Implications

**DOI:** 10.3389/fnins.2020.00021

**Published:** 2020-01-29

**Authors:** Chao Deng, Jeffrey K. Yao

**Affiliations:** ^1^Antipsychotic Research Laboratory, Illawarra Health and Medical Research Institute, Wollongong, NSW, Australia; ^2^School of Medicine and Molecular Horizons, University of Wollongong, Wollongong, NSW, Australia; ^3^Medical Research Service, VA Pittsburgh Healthcare System, Pittsburgh, PA, United States; ^4^Department of Psychiatry, University of Pittsburgh School of Medicine, Pittsburgh, PA, United States

**Keywords:** psychiatric disorders, schizophrenia, antipsychotic drugs, metabolic disorders, obesity, hyperlipidaemia, insulin resistance, diabetes

Accumulated evidence suggests that patients suffering with major psychotic disorders, such as schizophrenia, bipolar disorders, and major depression, have a higher risk of developing metabolic syndrome including weight gain, obesity, hyperlipidaemia, hyperglycaemia, and insulin resistance, which increase the risk of cardiovascular disease, stroke, and diabetes (Mitchell et al., 2011; Vancampfort et al., 2015). Although multiple factors have been proposed to explain the causes of these metabolic conditions/disturbances, the underlying mechanisms are still not fully understood. Among them, antipsychotic drugs, particularly the second generation (or atypical) antipsychotics, have been proven to be the largest contributors leading to such metabolic disorders (Deng, 2013; Reynolds and McGowan, 2017). However, there is longstanding debate on whether psychiatric disorders are the risk factors for the development of metabolic syndrome in these patients; apart from disease-related inactive lifestyle and poor dietary choices, it is not clear how much the pathophysiology of psychiatric disorders contributes to metabolic syndrome or *vice versa* (Vancampfort et al., 2015; Reynolds and McGowan, 2017; Lee et al., 2018). To date, there are only limited interventions in the prevention and treatment of metabolic disorders associated with antipsychotic medication in psychiatric patients.

This Frontier Research Topic has brought together a group of leading experts in the field to address these critical issues in four reviews, one perspective, one theoretic, and one original article. Three of these articles investigated how, and to what degree, the pathophysiology of psychiatric disorders bidirectionally interacts with metabolic dysfunction. Freyberg et al. have made an extensive search of evidence to answer the question—“*Is there an intrinsic metabolic risk inherent to schizophrenia or is the metabolic phenotype primarily driven by the impact of antipsychotics*,” based on data from the pre-antipsychotic era and more recently drug-naïve schizophrenia patients. They also proposed a model to explain the potential central and peripheral signaling pathways and molecules (such as dopamine, AKT1, and NRG1) linking the pathophysiology of schizophrenia and development of metabolic dysfunction, and antipsychotic-induced exacerbation of the pre-existing metabolic problems (Freyberg et al.). Consistent with Freyberg et al. and Castillo et al. presented further evidence supporting the shared common intrinsic susceptibility factors between schizophrenia and metabolic syndrome through alterations in sphingolipid metabolism. As major components of cell membranes and its participation in the formation of membrane “lipid rafts,” disturbance of sphingolipid homeostasis may lead to both neuropsychiatric disease and metabolic syndrome, therefore the authors proposed a specific sphingolipid profile from peripheral tissue (such as skin and blood) as biomarkers for schizophrenia and/or metabolic syndrome in an early schizophrenia diagnosis to help clinicians anticipate cardiometabolic complications in this patient group (Castillo et al.). On the other hand, Zuccoli et al. provided proteomic evidence from post-mortem brains indicating the energy metabolism dysfunction in the brain contributing to neuropathology underlying three major psychiatric disorders—schizophrenia, bipolar disorder, and major depressive disorder. It is interesting that similar alterations of five proteins involved in the main axis of metabolic pathways for ATP production are identified in all three disorders, while there are 32 altered proteins (mostly related to mitochondrial electron transport) shared between schizophrenia and bipolar disorders, 10 altered proteins (such as phosphoglucomutase 1) between schizophrenia and major depressive disorder, and 7 between bipolar disorder and major depressive disorder (Zuccoli et al.). Since the human brain accounts for only 2% of whole body weight, but utilizes over 20% of the body's energy, it is understandable that deficits in energy metabolisms could be a key pathophysiological aspect of these major psychiatric disorders.

Over the past decade studies from a number of laboratories have largely improved our understanding of the neuropharmacological and neuroendocrinological regulation of antipsychotic-induced weight gain and obesity that have been reviewed previously (Lian et al., 2016; Reynolds and McGowan, 2017). The following three papers explored the mechanisms for insulin resistance, dyslipidaemia, and diabetes associated with antipsychotic medication and potential intervention. del Campo et al. introduced a novel approach to study the alteration of the mitochondrial dynamics caused by olanzapine in an *in silico* experiment for revealing interactions between the insulin receptor and olanzapine. Their preliminary data suggested that a mitochondrial fission/fusion ration imbalance and inefficient mitochondrial phenotypes of muscle cells may lead to insulin resistance and hyperglycaemia (del Campo et al.). Chen et al. extensively reviewed the literature and summarized the findings into three mechanisms for antipsychotic-induced diabetes, in which the cellular signaling pathways responsible for insulin resistance due to the direct effect of antipsychotics (particularly olanzapine and clozapine) or indirectly via antipsychotic-induced obesity were clearly elucidated. Furthermore, it has been found that antipsychotics can also decrease insulin secretion by damaging pancreatic β-cells via direct (mediated by ATP) and indirect (e.g., acting at the muscarinic M3 receptor) signaling pathways, as well as activating apoptosis pathways, that may also be the mechanisms underlying antipsychotic-induced hyperglycaemic emergency (Chen et al.). It has been reported previously that olanzapine and clozapine-induced lipid disturbance via AMP-activated protein kinase (AMPK) pathways (Liu et al., 2015). Liu et al. explored potential intervention to prevent dyslipidaemia via activating hepatic AMPK pathways in a chronic rat model. Curcumin, a major active compound of *Curcuma longa*, was identified as a novel AMPK agonist and able to alleviate the clozapine-induced dyslipidaemia via mediating the AMPA-SREBPs pathways (Liu et al.).

Finally, there is a lasting debate on whether antipsychotic-induced weight is a completely negative side-effect, or whether it has some therapeutic benefits in the treatment of psychiatric disorders. To address this issue, Raben et al. reviewed 31 independent studies including 6,063 subjects being treated with various antipsychotics ranging from 4 to 384 weeks to identify associations between weight gain and symptom improvement. Their findings support an association between antipsychotic-induced weight gain and therapeutic benefit in schizophrenia patients, particularly in those treated with olanzapine and clozapine (both with the most pronounced effects on insulin dysregulation), suggesting insulin regulation as a possible explanation (Raben et al.).

In summary, studies published in this Research Topic provided a picture of complex bidirectional interactions between the pathophysiology of major psychiatric disorders, metabolic dysfunction, and antipsychotic medication. Deficits in some common signaling pathways (e.g., AKT1, insulin regulation and energy metabolism) may contribute to both intrinsic and antipsychotic-induced metabolic dysfunction, as well as the pathophysiology of these psychiatric disorders. Although a correlation between antipsychotic-induced weight gain and therapeutic benefit has been reported, current evidence does not support weight gain as necessary for therapeutic benefit (Raben et al.). Therefore, early diagnosis and prevention of metabolic dysfunction are important and critical for the management of antipsychotic medication in psychiatric patients. We believe that this Frontier Research Topic will stimulate additional studies to take innovative approaches for further revealing interactive neuropathogenetic mechanisms of psychiatric disorders and metabolic disorders, and to identify novel targets of the pathophysiology of psychiatric disorders with limited metabolic side-effects, while more efforts in the development of pharmacological and non-pharmacological interventions preventing/treating metabolic disorders in psychiatric patients are also important.

## Author Contributions

CD drafted the manuscript. During the course of this Research Topic, JY passed away suddenly. JY has made significant contributions to all aspects of this Research Topic, from forming the idea of the Topic proposal to inviting contributing authors and editing the manuscript.

### Conflict of Interest

The authors declare that the research was conducted in the absence of any commercial or financial relationships that could be construed as a potential conflict of interest.
